# Lamivudine plus tenofovir combination therapy versus lamivudine monotherapy for HBV/HIV coinfection: a meta-analysis

**DOI:** 10.1186/s12985-018-1050-3

**Published:** 2018-09-10

**Authors:** Aoran Luo, Xiaoyan Jiang, Hong Ren

**Affiliations:** Key Laboratory of Molecular Biology for Infectious Diseases (Ministry of Education), Institute for Viral Hepatitis, Department of Infectious Diseases, The Second Affiliated Hospital, Chongqing Medical University, Chongqing, People’s Republic of China

**Keywords:** Lamivudine, Tenofovir, HBV/HIV coinfection

## Abstract

**Background:**

Currently, there is no consensus on the efficacy and safety of lamivudine (LAM) plus tenofovir disoproxil fumarate (TDF) combination therapy versus lamivudine monotherapy in HBV/HIV coinfected patients.

**Methods:**

A comprehensive literature search was performed in English and Chinese databases. Both relevant dichotomous and continuous variables were extracted, and the combined outcomes were expressed as a risk ratio (RR) or a standard mean difference (SMD).

**Results:**

Eleven eligible studies were included in our analysis. For HBV-relevant outcomes, the proportion of patients with undetectable HBV, the rates of serum alanine aminotransferase (ALT) normalization and hepatitis B e antigen (HBeAg) loss were higher in the combination therapy group than the monotherapy group (RR = 1.42, 95% CI: 1.14–1.76, *P* = 0.002; RR = 1.36, 95% CI: 1.17–1.58, *P* < 0.0001; RR = 2.74, 95% CI: 1.20–6.22, *P* = 0.02). In addition, the rate of HIV RNA-negative conversion was higher in the combination therapy group than the monotherapy group (RR = 1.26, 95% CI: 1.11–1.42, *P* = 0.0003).

**Conclusion:**

LAM plus TDF combination therapy was more efficacious than LAM monotherapy in HBV/HIV coinfected patients. As time passes, this difference becomes more pronounced.

**Electronic supplementary material:**

The online version of this article (10.1186/s12985-018-1050-3) contains supplementary material, which is available to authorized users.

## Background

Because HIV and HBV infection share common routes of transmission, the prevalence of hepatitis B markers (anti-HBc, anti-hepatitis B core antibodies and/or HBs-Ag, the hepatitis B surface antigen) is up to 90% among HIV-infected patients. Among the estimated 40 million patients who are infected with HIV around the world, approximately 2–4 million are chronically infected with HBV [1]. Currently, the available antiviral drugs for chronic hepatitis B include interferon-alpha and nucleos(t)ide analogues (lamivudine, telbivudine, and entecavir, adefovir and tenofovir), which are HBV polymerase inhibitors [2]. These nucleos(t)ide analogs, such as lamivudine (LAM) and tenofovir disoproxil fumarate (TDF) are also employed for HIV treatment. Lamivudine was approved by the Food and Drug Administration (FDA) in November of 1995 [3]. TDF received approval for treating HIV infections from the FDA in October of 2001 and approval for the treatment of chronic HBV infection in August of 2008 [4, 5].

Despite the fact that the guidelines now recommend TDF and LAM or emtricitabine in combination as a preferred therapy for patients with HIV/HBV coinfections [6], the high prevalence areas of HIV/HBV coinfection are primarily concentrated in less developed regions. For example, Western African countries have the highest coinfection rates (median: 11.5%) in the world [7, 8]. TDF is not yet widely available or used in these places due to more expensive than LAM. Therefore, LAM is more affordable than TDF for the patients in these areas.

A recent meta-analysis only combined the rate of HBV responses in HBV/HIV coinfected patients who were treated with TDF, but it did not compare the LAM plus TDF combination therapy with LAM monotherapy because of a lack of relevant clinical trial reports [9]. In addition, recent relevant clinical studies were not consistent. A meta-analysis is a useful quantitative method for combining the results from multiple studies, especially when the results are not consistent. Above all, the aim of the present study was to conduct a meta-analysis of existing trials to compare the TDF plus LAM combination therapy with lamivudine monotherapy in HBV/HIV coinfected patients.

## Methods

### Search strategy

Searches of English-language databases (PubMed, EMBASE, Web of Science, the Cochrane Library, and ClinicalTrials.gov) and Chinese databases [China National Knowledge Infrastructure (CNKI), the Chinese BioMedical Literature Database (CBM), WanFang, and the Chinese Science and Technology Periodicals Database (VIP)] from their inception to March 2018 were conducted by two investigators (Aoran Luo and Xiaoyan Jiang). Reference lists of all the retrieved articles were manually searched for potentially relevant reports that were missed by the intelligent retrieval systems. The search strategy was as follows: ((((tenofovir OR “9-(2-Phosphonylmethoxypropyl)adenine” OR viread OR apropovir OR truvada OR atripla OR pmpa)) AND (lamivudine OR “2,3-Dideoxy-3-thiacytidine” OR “2,3 Dideoxy 3 thiacytidine” OR “3TC” OR Epivir OR “lamivudine, (2S-cis)-Isomer” OR “BCH-189” OR “BCH 189” OR “GR-109714X” OR “GR109714X”)) AND (HBV OR “hepatitis b”)) AND (HIV OR “human immunodeficiency virus” OR AIDS).

### Selection criteria

Published studies comparing the efficacy of LAM monotherapy to LAM plus TDF combination therapy in HBV/HIV coinfection were included in our meta-analysis, if the study was eligible and the data were available. The inclusion criteria were as follows: (1) Study design: RCTs, with retrospective and prospective cohort study designs (each group sample size > 10); (2) Subjects: Adult patients with HBV/HIV coinfection; (3) Treatment strategy: including a de novo LAM (100 mg/day) plus TDF (300 mg/day) combination therapy group and a LAM (100 mg/day) monotherapy group. (4) If, in addition to LAM and TDF the two groups used the same other drugs, those studies were also included. (5) Outcome: including virological responses such as rates of undetectable HBV DNA and HBV DNA levels at the end of the treatment, the rates of HBeAg loss and HBeAg seroconversion; Biochemical response such as ALT levels, and rates of ALT normalization; OR including HIV outcomes including levels of HIV RNA, CD4^+^ T cells, etc. The exclusion criteria were as follows: (1) duplicated data; (2) coinfection with hepatitis A, C, D, or E viruses; and (3) autoimmune hepatitis, alcoholic liver disease, primary biliary cirrhosis, Wilson’s disease, hepatocellular carcinoma, etc. (4) If, in addition to LAM and TDF, the two groups used other different drugs, those studies were also excluded; and (5) any report for which sufficient information was not available.

### Outcome measures

The primary efficacy end-point was the rate of HBV virological response, which was defined as the proportion of patients with undetectable serum HBV-DNA after treatment. The secondary end points were as follows: the end-of-treatment HBV DNA level, HBeAg loss rate; biochemical response, as defined as the normalization of ALT; HIV outcomes including the HIV RNA-negative conversion ratio, which was defined as the proportion of patients with undetectable serum HIV-RNA after treatment; and the levels of CD4^+^T cells.

### Study quality assessment

The revised Jadad quality scale had a maximum number of 7 points, and it was used to assess the quality of all 9 RCTs by examining the description of the randomization method, blinding method, description of deviations and drop-outs. Out of the 9 RCTs, only one received a Jadad score of 4, and the Jadad scores were 2 for six studies, and 3 for the remaining 2 studies. All 9 studies claimed to be RCTs, but only 3 studies reported the randomization method, and none of the studies was blinded. Only one study described the study withdrawals and dropouts in detail.

The Newcastle-Ottawa Scale (NOS) was used to assess the two included retrospective cohort studies based on several standards including the selection of cohorts, comparability of cohorts, and the assessment of the outcomes. Out of the two retrospective cohort studies, one received an NOS score of six and the other one received a seven.

### Data extraction

All the data were independently extracted from the included studies by two investigators (Aoran Luo and Xiaoyan Jiang), and two authors (Aoran Luo and Xiaoyan Jiang) independently assessed the retrieved reports according to the inclusion/exclusion criteria. Any dispute between investigators was resolved through arbitration by a third party (Hong Ren). The data were extracted for their (1) study characteristics (author and year of publication, geographic locale, study design, and sample size); (2) patient demographics (age, sex, and HBeAg-positive percentage) and baseline characteristics (alanine aminotransferase, serum HBV DNA levels, and CD4^+^T cell); (3) treatment details (anti-viral agent used, dose of drug, and duration of treatment); and (4) the study outcomes at the end of the treatment.

### Statistical analysis

Both the dichotomous and continuous variables were extracted during this meta-analysis. For the dichotomous outcomes, the results were presented as the relative risk (RR) with a 95% confidence interval (95% CI), while the continuous results were presented as a standardized mean difference (SMD) with a 95% confidence interval (95% CI). The statistical heterogeneity was assessed by using chi-square and I-square (I^2^) tests with significance set at *p* < 0.1. If the I^2^ value exceeded 50%, then the random effect model was used on combined results. Otherwise, the fixed effect model was used. To investigate the source of heterogeneity, the Galbraith plot was employed to spot the outliers as the possible major sources of heterogeneity; a sensitivity analysis was then performed through the sequential omission of individual studies to investigate the effect of each study on the heterogeneity. In addition, a meta-regression was used to assess the effect of the treatment time on the outcomes. The possible publication bias was assessed by Funnel plot and Begg’s and Egger’s tests [10, 11].

All the statistical analyses were performed with Review Manager Software 5.3 (Cochrane Collaboration, Oxford, UK) and Stata (version 12.0). All the *p* values were two-tailed. Apart from Cochran’s Q-test, all tests with a *p* value < 0.05 were considered statistically significant.

## Results

### Search results and study characteristics

**Fig. 1:**
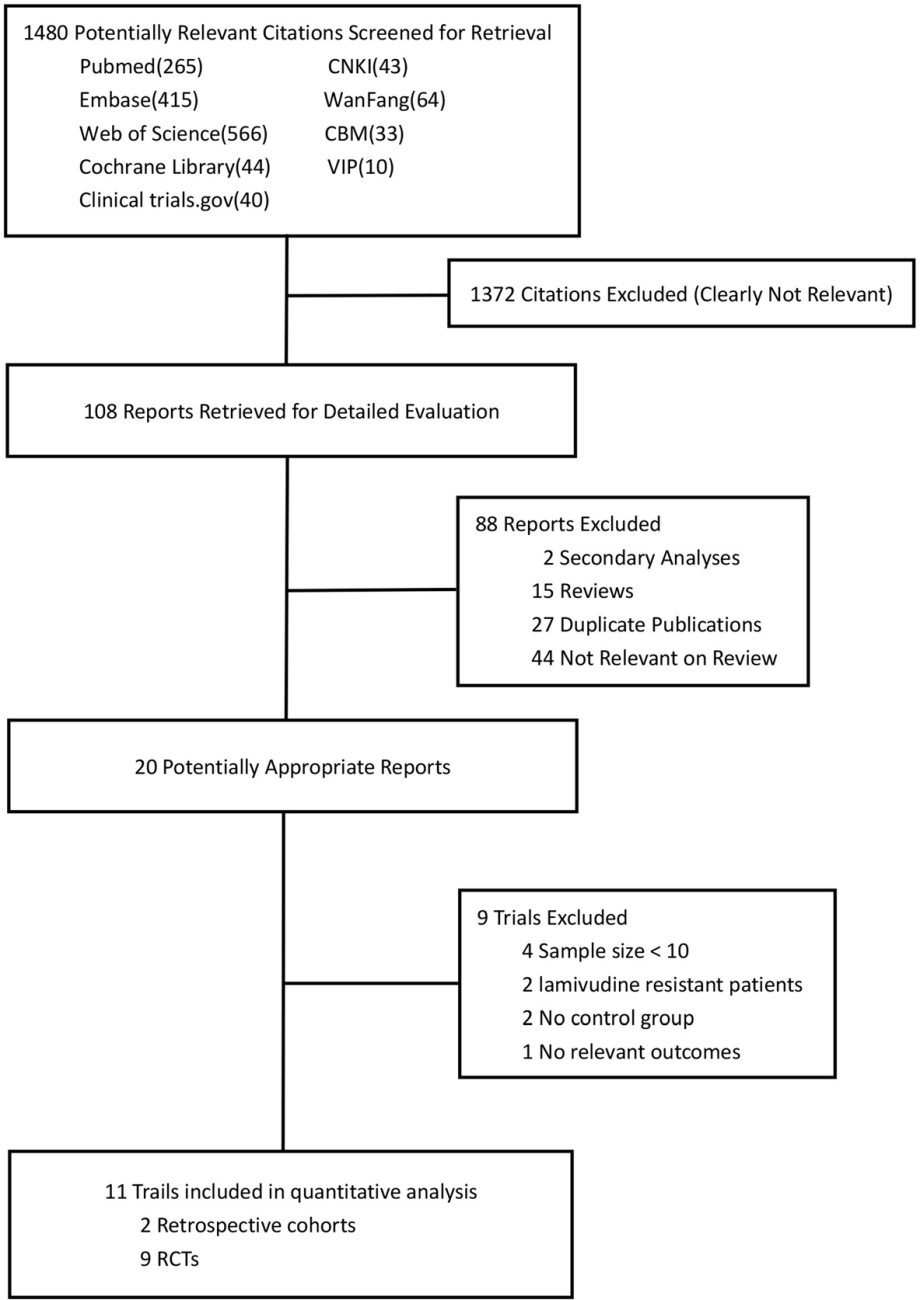
Study selection process

Initially, 1480 publications were identified in our database searches. After an initial screening, 1372 studies were clearly not relevant. The remaining 108 reports were retrieved for detailed evaluation. 88 studies were then excluded, two of which were secondary analyses, 15 were review articles, 27 were duplicate publications, and 44 were not relevant. Among the remaining 20 potentially appropriate reports, nine trials were excluded, four of which were due to small sample sizes, and the patients in two studies were lamivudine-resistant. There was no control group in two studies, and there were no relevant outcomes in one study. Lastly, 11 studies[12–22]were included in this meta-analysis, which consisted of 827 patients. Figure 1 shows the study selection process.

As summarized in Tables 1 and 2, the included studies were published between 2013 and 2017. Ten of 11 studies were from different provinces of China, and one was from the USA. Among the eleven studies, three were published in English, and the others were published in Chinese. Two studies were retrospective cohorts and the other nine studies were randomized controlled trials (RCTs). The sample size for each of the studies ranged from 25 to 151. The mean age ranged from 29 to 54 years old. The percentage of males ranged from 0 to 84.2%.

**Table 1:** Characteristics of the included trials in this meta-analysis

Author	Year	Geographic Locale	Study Design	Regimen	Sample size	Duration, weeks
Chen [15]	2017	China,Sichuan province	RCT	TDF:300 mg/d; LAM:100 mg/d	100	48
Chen [16]	2013	China,Jiangsu province	RCT	TDF:300 mg/d; LAM:100 mg/d	60	72
Gao [17]	2016	China,Jilin province	RCT	TDF:300 mg/d; LAM:100 mg/d	70	48
He [18]	2015	China,Guangdong province	RCT	TDF:300 mg/d; LAM:100 mg/d	50	48
Jain [12]	2006	the USA	Retrospective cohort	TDF:300 mg/d; LAM:100 mg/d	25	48
Li [13]	2016	China,multi-centers	Retrospective cohort	TDF:300 mg/d; LAM:100 mg/d	151	48
Liu [19]	2017	China,Shaanxi province	RCT	TDF:300 mg/d; LAM:100 mg/d	120	72
Luo [20]	2016	China,Jiangxi province	RCT	TDF:300 mg/d; LAM:100 mg/d	20	24
Min Zhou [21]	2016	China,Jiangxi province	RCT	TDF:300 mg/d; LAM:100 mg/d	90	48
Wang [14]	2016	China,Guangxi province	RCT	TDF:300 mg/d; LAM:100 mg/d	31	11~ 24
Yan Zhou [22]	2016	China,Henan province	RCT	TDF:300 mg/d; LAM:100 mg/d	110	48

Sample size and duration were expressed in mean

**Table 2:** Characteristics of the included patients in this meta-analysis

Author	Year	Age	Sex(male%)	HBV DNA(log10)	HBeAg(+), %	ALT, U/L	CD4^+^T Cell/μl
Chen [15]	2017	42	55	5.02	NR	NR	NR
Chen [16]	2013	38	82	5.6	58.3	246	100
Gao [17]	2016	37	58.6	5.03	NR	NR	NR
He [18]	2015	39	64	5.02	NR	NR	NR
Jain [12]	2006	39	84.2	7.8	76	60	180
Li [13]	2016	36	82.8	3.59	25.8	29	200
Liu [19]	2017	31	59	NR	34.1	NR	NR
Luo [20]	2016	54	65	NR	15	NR	41
Min Zhou [21]	2016	36	51	NR	NR	NR	NR
Wang [14]	2016	29	0	3.83	29	NR	322
Yan Zhou [22]	2016	38	57.2	5.04	NR	NR	NR

HBV DNA, HBeAg, ALT, and CD4^+^T cell were all expressed in mean. NR: not report

### Comparison of HBV-related outcomes in LAM plus TDF combination therapy patients and LAM monotherapy patients

**Fig. 2:**
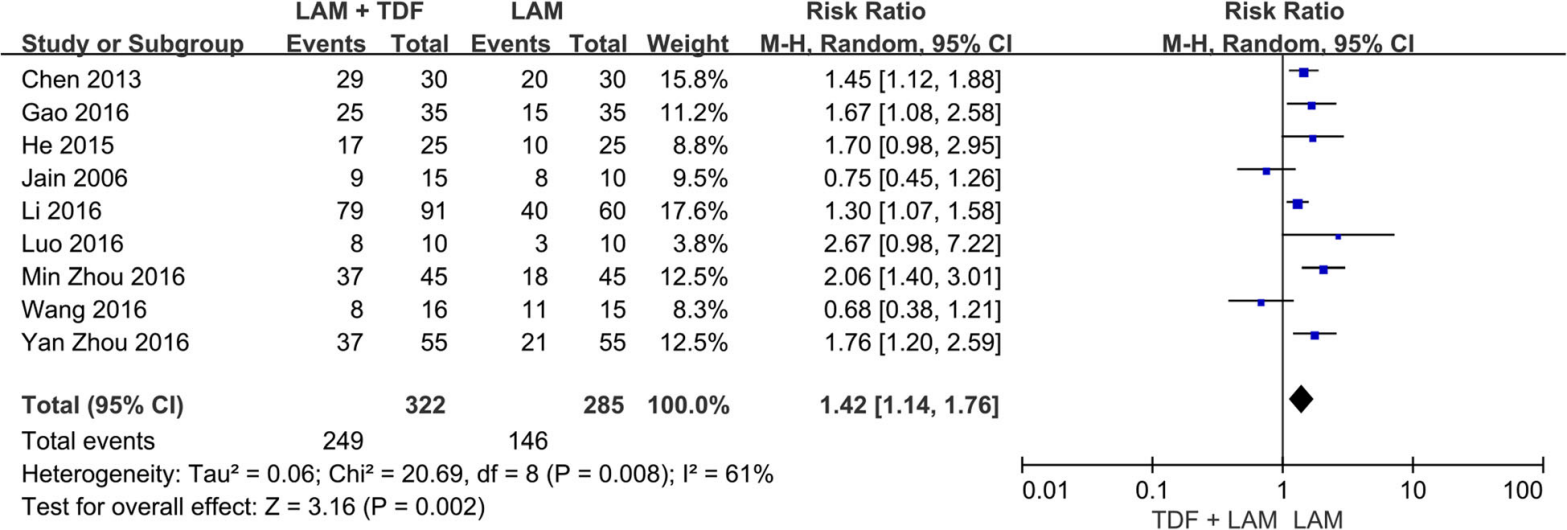
Effect of LAM + TDF vs. LAM on HBV virologic response

The nine included studies, which involved 607 patients, reported the undetectable rates of HBV DNA [12–14, 16–18, 20–22]. Because there was significant heterogeneity among these studies (*P* = 0.008, I^2^ = 61%), the random-effect method was applied to calculate the overall effects. The rate of undetectable HBV DNA was higher in the combination therapy group than in the monotherapy group (RR = 1.42, 95% CI: 1.14–1.76, *P* = 0.002; Fig. 2).

In the subgroup analyses by language, the results suggested that for studies published in Chinese, the rate of undetectable HBV DNA was significantly higher in the combination therapy group than in the monotherapy group (RR = 1.55, 95% CI: 1.25–1.93, *P* < 0.0001; Additional file 1: Figure S1), while the rate was similar between the two groups for studies published in English (RR = 0.93, 95% CI: 0.58–1.48, *P* = 0.75; Additional file 1: Figure S1). In the subgroup analyses by study design, the combined results showed that for randomized controlled trials, the rate of undetectable HBV DNA was significantly higher in the combination therapy group than in the monotherapy group (RR = 1.57, 95% CI: 1.23–2.00, *P* = 0.0002; Additional file 2: Figure S2), and the rate was similar between the two groups for cohort studies (RR = 1.04, 95% CI: 0.61–1.77, *P* = 0.88; Additional file 2: Figure S2). The subgroup analyses by area suggested that for studies conducted in the southern regions of China, the rate of undetectable HBV DNA was significantly higher in the combination therapy group than in the monotherapy group (RR = 1.50, 95% CI: 1.04–2.17, *P* = 0.03; Additional file 3: Figure S3), while the rate was similar between the two groups for studies conducted in the northern regions of China (RR = 1.02, 95% CI: 0.81–1.28, *P* = 0.87; Additional file 3: Figure S3).

**Fig. 3:**
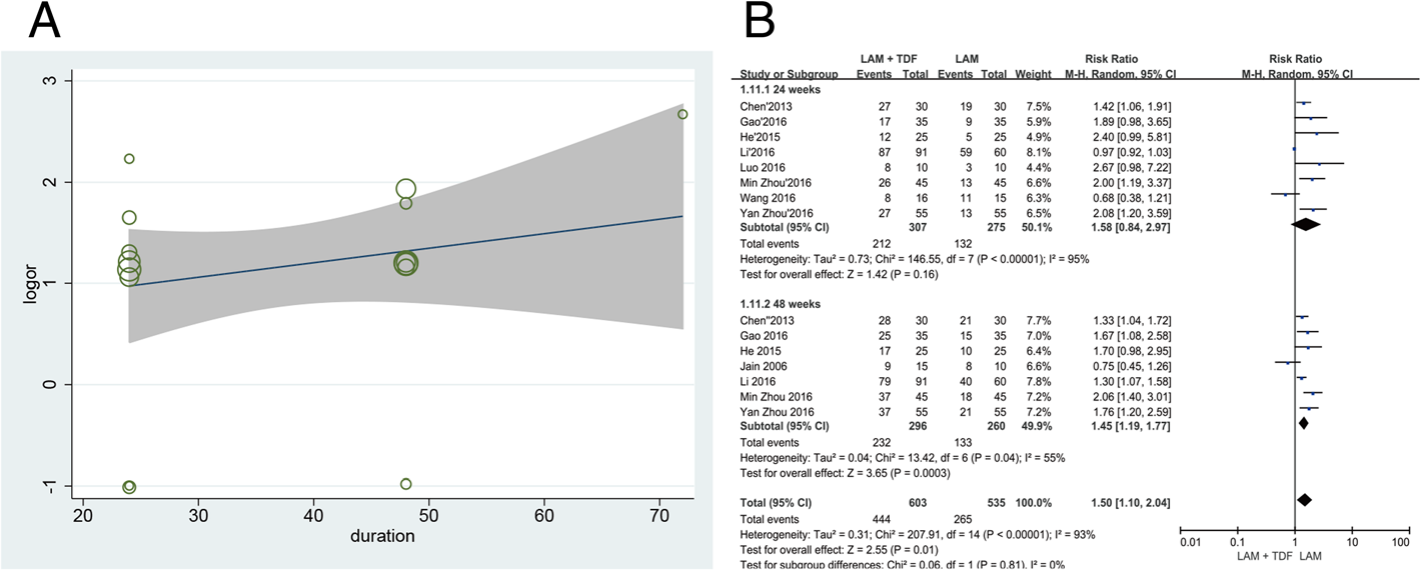
Meta-regression of treatment duration (**a**), and subgroup analyses by 570 treatment duration (**b**)

In addition, we also used meta-regression to detect whether the treatment duration affects the virological response rate. Although the regression diagram indicates that the logOR between the two groups increased with time, the increase is not statistically significant (*P* = 0.322; Fig. 3a). This finding may be due to the lack of sufficient data for 72 weeks, so we also compared the data between 24 and 48 weeks. The results showed that for 24-week studies, the virological response rate was similar between the two groups (RR = 1.58, 95% CI: 0.84–2.97, *P* = 0.16; Fig. 3b), but the rate of undetectable HBV DNA was significantly higher in the combination therapy group than in the monotherapy group for 48-week studies (RR = 1.45, 95% CI: 1.19–1.77, *P* = 0.0003; Fig. 3b). Therefore, over time, the effect of the combination therapy was better.

Six studies including 540 patients reported the serum HBV DNA levels at the end of the therapy [15, 17–19, 21, 22]. Because the HBV DNA levels are comparable in these studies at the baseline, the difference in therapeutic effects can be illustrated by comparing the HBV DNA level of the treatment endpoint. Because there was significant heterogeneity among these studies (*P* < 0.00001, I^2^ = 98%), the random-effects model was used to pool the results. The meta-analysis showed that the HBV DNA levels were similar between the two groups (SMD = − 0.86, 95% CI: -2.53–0.81, *P* = 0.31; Additional file 4: Figure S4).

The two included studies involving 132 patients reported the rates of ALT normalization [15, 16]. The between-study heterogeneity was not significant when the 2 studies were pooled into a meta-analysis (*P* = 0.86, I^2^ = 0%); thus, the fixed-effects model was used to pool the results. The results of pooling the 2 studies showed that the rate of ALT normalization was higher in the combination therapy group than in the monotherapy group (RR = 1.36, 95% CI: 1.17–1.58, *P* < 0.0001).

The three included studies involving 87 patients reported the rates of HBeAg loss [12, 16, 19]. The between-study heterogeneity was not significant when the 3 studies were pooled into a meta-analysis (*P* = 0.46, I^2^ = 0%); thus, the fixed-effects model was used to pool the results. The results suggested that the rate of HBeAg loss was higher in the combination therapy group than in the monotherapy group (RR = 2.74, 95% CI: 1.20–6.22, *P* = 0.02; Additional file 5: Figure S5).

### Comparison of HIV-related outcomes in LAM plus TDF combination therapy patients and LAM monotherapy patients

**Fig. 4:**
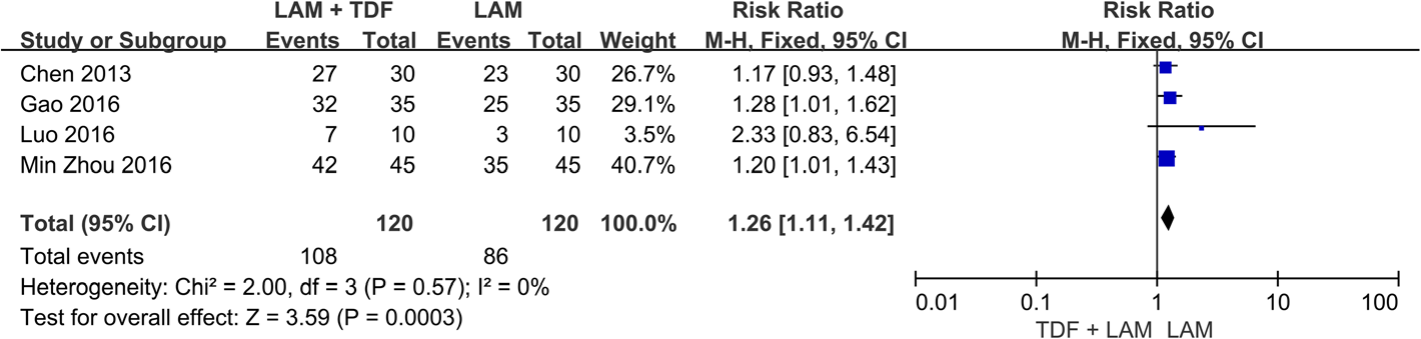
Effect of LAM + TDF vs. LAM on HIV RNA Negative Conversion Rate

The four included studies involved 240 patients and their reported rates of HIV RNA-negative conversion [16, 17, 20, 21]. The between-study heterogeneity was not significant when the 4 studies were pooled into a meta-analysis (*P* = 0.57, I^2^ = 0%); thus, the fixed-effects model was used to pool the results. The meta-analysis showed that the rate of HIV RNA-negative conversion was higher in the combination therapy group than in the monotherapy group (RR = 1.26, 95% CI: 1.11–1.42, *P* = 0.0003; Fig. 4).

Two of the included studies involving 140 patients reported the levels of CD4^+^ T cells at the end of the treatment [19, 20]. Because there was significant heterogeneity among these studies (*P* < 0.00001, I^2^ = 96%), the random-effect method was applied to calculate the overall effects. The result showed that the levels of CD4+ T cells at the end of the treatment were similar between the two groups (SMD = 2.06, 95% CI: -0.64–4.77, *P* = 0.14).

### Safety comparison in LAM plus TDF combination therapy patients and LAM monotherapy patients

The two studies included here reported some of the adverse events that occurred over the course of treatment, including dizziness, nausea, myelosuppression, constipation and elevated blood lipids [19, 20]. The between-study heterogeneity was significant when the 2 studies were pooled into the meta-analysis (*P* = 0.10, I^2^ = 62%); thus, the random-effects model was used to pool the results. The meta-analysis showed that the incidence of adverse events was similar between the two groups (RR = 0.72, 95% CI: 0.18–2.94, *P* = 0.65).

### Heterogeneity analysis and sensitivity analysis

The between-study heterogeneity was significant in the pooling analyses for some outcomes (the *P* values for the HBV virological response rate, serum HBV DNA levels, CD4+ T Cells, and Safety were all less than 0.10 and the I^2^ values were 61%, 98%, 96%, and 62% respectively). Furthermore, the between-study heterogeneity was also significant in all the subgroup analyses apart from the subgroup analyses in northern China. For the HBV virological response rate, the subgroup analysis showed that heterogeneity comes from different publishing languages, research designs, areas and treatment times. However, as illustrated in Additional file 6: Figure S6, the Galbraith plots spotted two studies (Wang2016,Jain2006)[12, 14]as the outliers and the possible major sources of heterogeneity. Coincidentally, when Galbraith plots were used to analyze the heterogeneity of the subgroups, the heterogeneity was also primarily from one of these two studies. For the serum HBV DNA levels, Galbraith plots were used to spot two studies (Min Zhou2016,Liu2017)[19, 21]as the outliers and the possible major sources of the heterogeneity. For the HBV virological response rate, the serum HBV DNA levels and all the subgroup analyses except the 24-week subgroup, the heterogeneity was adjusted and eliminated (all their *P* values were higher than 0.10) after excluding the outliers as the possible major source of heterogeneity. However, the heterogeneity in the analyses of the 24-week subgroup was not significantly adjusted (the *P* values were all lower than 0.10) by excluding the one study(Wang2016) [14]that was spotted as the outlier by the Galbraith plot method. Therefore, the sensitivity analysis was performed through the sequential omission of the other individual studies except for Wang 2016. It turned out that the heterogeneity was adjusted and eliminated (*P* = 0.49, I^2^ = 0%) after excluding the Wang2016 and Li2016 studies. Additionally, for any other analyses that pooled more than three individual studies, the significance of the RRs was not influenced excessively by omitting any single study except for outliers spotted by Galbraith plots (data not shown).

### Publication bias

Funnel plots and Egger’s and Begg’s tests were performed to evaluate the publication bias of the studies on the HBV virological response in this meta-analysis. As shown in Additional file 7: Figure S7, the Funnel plot shapes did not reveal obvious evidence of asymmetry, and all the *p* values of the Egger’s and Begg’s tests were greater than 0.05, providing statistical evidence of the funnel plot symmetry. Thus, the results above suggested that the publication bias was not evident in this meta-analysis.

In addition, we were unable to assess the publication bias due to the small number of studies for other outcomes.

## Discussion

For hepatitis B virus or HIV infection alone, compared to the other four nucleoside analogues,TDF has more effective virus suppression, higher drug resistance barrier, and good safety. Therefore, TDF is recommended as a first-line treatment for hepatitis B virus hepatitis and HIV infection alone. However, in response to HBV/HIV coinfection, related studies have found that TDF monotherapy was not more effective than TDF in combination with LAM or emtricitabine [23], so the guidelines recommend TDF in combination with LAM or emtricitabine to treat HBV/HIV coinfection [24]. Although the guidelines explicitly recommend the use of TDF-based combination therapy for HBV/HIV coinfected patients [6], there are relatively few large clinical trials for comparing between TDF-based combination therapy and LAM monotherapy. Additionally, the results of the existing clinical trials are not consistent, so a meta-analysis is necessary. A previous meta-analysis of 23 studies only showed the overall proportion of HBV replication suppression achieved by TDF treatment in HBV/HIV coinfected patients [9]. Due to the limited number of studies, it did not compare the TDF and LAM combination therapy with LAM monotherapy. Some new studies have emerged in recent years, so we performed this meta-analysis to combine the results.

This meta-analysis was performed by carefully reviewing 9 individual RCT studies and 2 retrospective cohort studies to compare between LAM plus TDF combination therapy and LAM monotherapy in HBV/HIV coinfected patients. Subgroup analyses for the HBV virological response rate were primarily addressed through the study design, publishing language, or by the area in southern and northern China. In addition, a heterogeneity analysis and a sensitivity analysis were implemented to ensure the epidemiological credibility of this meta-analysis.

We found that for the HBV outcomes (HBV suppression, ALT normalization rate, and HBeAg loss rate) and HIV RNA-negative conversion ratio, the LAM plus TDF combination therapy was more effective than LAM monotherapy. However, this trend was not significant for the numbers of CD4+ T cells at the end of the treatment and the rate of adverse events over the course of the treatment owing to the limited relevant studies included in this meta-analysis.

In all the subgroup analyses on the HBV virological response rate, there were always significant differences between the two groups in the subgroups, while the difference was not significant in the subgroup that included no more than three studies. Therefore, these subgroups may have insufficient statistical power to detect a slight effect or they may have generated a fluctuation risk estimate. When stratified for areas, there was a significant difference between the two groups in southern China, but the difference was not significant in northern China. This trend not only occurred because of the different living habits of the two places, but more likely because the HBV genotype distribution is different. The predominant HBV genotype in southern China is genotype B, and genotype C is more prevalent in the northern part of China. Some clinical studies have found that genotype B infection (23%) has a better response rate than genotype C infection (11%) [25]. The mechanisms of these observed differences are unclear, but may include the following reasons: (1) sensitivity of the polymerase during treatment; (2) natural differences in the immune system’s elimination of differences in specific HBV genotypes; (3) BCP (basal core promoter) mutations in HBV genotypes A and C are often higher than those in D and B genotypes, while BCP mutations are associated with promoting viral replication, poor response to treatment and advanced liver disease [26, 27].

In addition, we also used meta-regression and subgroup analysis to detect whether the treatment duration affects the virological response rate. The results suggested that over time, the effect of the combination therapy was better. On one hand, there is a high frequency of resistance mutations, even after a short-course treatment of LAM. The common drug resistant mutations include tyrosine-methionine-aspartate-aspartate mutations known as the YMDD mutant [28, 29]. On the other hand, TDF could inhibit virus replication, with a high barrier to drug resistance [2]. Thus, it is reasonable that with the extended treatment duration, the effect of the combination therapy was better than that of LAM monotherapy.

Although LAM/TDF combination therapy has the following advantages over LAM monotherapy: (1) Obviously, as the results of this study showed, combination therapy can inhibit the virus more effectively; (2) Compared to LAM monotherapy, combination therapy is less likely to produce viral resistance; (3) patients who use combination therapy are less likely to have viral breakthroughs; (4) Because TDF has good safety, combination therapy does not increase the incidence of adverse events. The potentially increased risk of toxicity must always be noted when instituting LAM and TDF combinations. Although the incidence of adverse events was similar between the two groups in the present meta-analysis, the number of studies included here may be too few to show a significant difference. Renal and bone toxicity were rarely reported in CHB patients [30], but they are associated with TDF in HIV-infected patients [31]. Therefore, the increased risk of toxicity must be additionally monitored for HBV/HIV coinfected patients.

Heterogeneity is an underlying problem when elucidating the results of all the meta-analysis, identifying the sources of heterogeneity is one of the most important aims of a meta-analysis. In this meta-analysis, we evaluated the between-study heterogeneity by using four different methods, including the chi-square-based Q statistical test (Cochran’s Q statistic) [32] to test for heterogeneity, the I^2^ statistic to quantify the heterogeneity [33], Galbraith plots to identify outliers as the possible major sources of heterogeneity [34], and a sensitivity analysis to test the effect of each study on the heterogeneity. The result showed that the heterogeneity of the present study was primarily derived from these two studies (Jain2006 and Wang2016) [12, 14]. The biggest differences between these two studies and others are the patients. The patients included in Jain2006 were all from the USA, while the patients included in other studies were all from China; the patients included in Wang2016 were all pregnant women. Estrogen is at its peak during pregnancy. Estrogens are at their peak during pregnancy and have the opposite effect on HBV replication compared to androgen. Specifically, the androgen and estrogen pathways were confirmed to counter-regulate HBV transcription by targeting viral enhancer I. In HBV biology, the androgen-activated androgen receptor actively binds to viral enhancer I and stimulates viral transcription comprehensively, while estrogen-sustained estrogen receptor α passively eliminates hepatocyte nuclear factor 4α from activating enhancer I and then restraining HBV transcription [35].

Some possible limitations within this meta-analysis should be acknowledged. First, there was too little detailed information on individual patients to evaluate the treatment effects in the different subgroups. Second, nine randomized controlled trials and two retrospective cohorts were included, so not all of the included studies were randomized controlled trials. Third, because the included patients were almost all Chinese, the results may not extend to other populations around the world. Fourth, as the revised Jadad quality scale showed, the quality of the randomized controlled trials included here was not very high. Fifth, because the number of studies included here varied for each outcome, the statistical power was sometimes too small to detect the treatment effects, especially when the number of included studies was low.

Despite these limitations, the present study is the first meta-study to show that LAM plus TDF combination therapy is more effective than LAM monotherapy for HBV suppression in HBV/HIV coinfected patients. However, significant observations were found primarily for Chinese but not for other populations, so large and carefully designed studies from around the world are needed to provide the best evidence for these conclusions.

## Conclusions

LAM plus TDF combination therapy is more effective than LAM monotherapy for HBV suppression in HBV/HIV coinfected patients.

## Additional files

Additional file 1: **Figure S1.** Subgroup analyses by language. (TIF 7780 kb)

Additional file 2: **Figure S2.** Subgroup analyses by study design. (TIF 7777 kb)

Additional file 3: **Figure S3.** Subgroup analyses by areas of China. (TIF 7222 kb)

Additional file 4: **Figure S4.** Effect of LAM + TDF vs. LAM on HBV DNA levels at the end of treatment. (TIF 3430 kb)

Additional file 5: **Figure S5.** Effect of LAM + TDF vs. LAM on the rate of HBeAg loss. (TIF 1169 kb)

Additional file 6: **Figure S6.** Galbraith plots of HBV virological response rate(A), levels of HBV DNA(B), subgroups analysis of southern China(C), RCTs(D), 24 weeks(E), and 48 weeks(F). (TIF 6228 kb)

Additional file 7: **Figure S7.** Funnel plot for studies included for HBV virological responses. (TIF 516 kb)
