# Supplementary figures and images for: Lamivudine plus tenofovir combination therapy versus lamivudine monotherapy for HBV/HIV coinfection: a meta-analysis
Source: Virol J. 2018 Sep 10;15:139. doi: 10.1186/s12985-018-1050-3 (PMC6130076; doi:10.1186/s12985-018-1050-3)

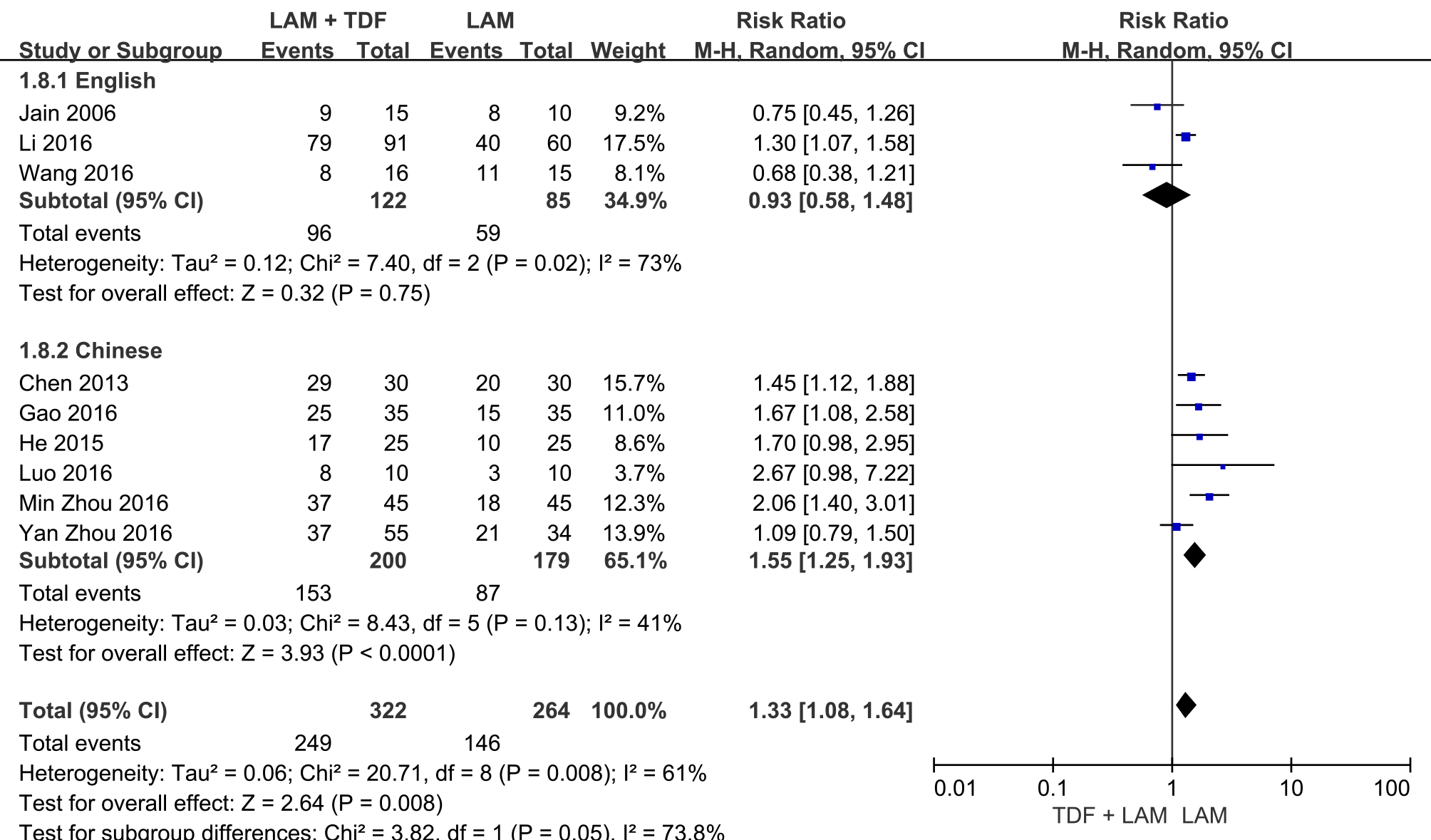

Supplement: Supplementary file 1 — Figure S1. Subgroup analyses by language. (TIF 7780 kb) [file 12985_2018_1050_MOESM1_ESM.tif]

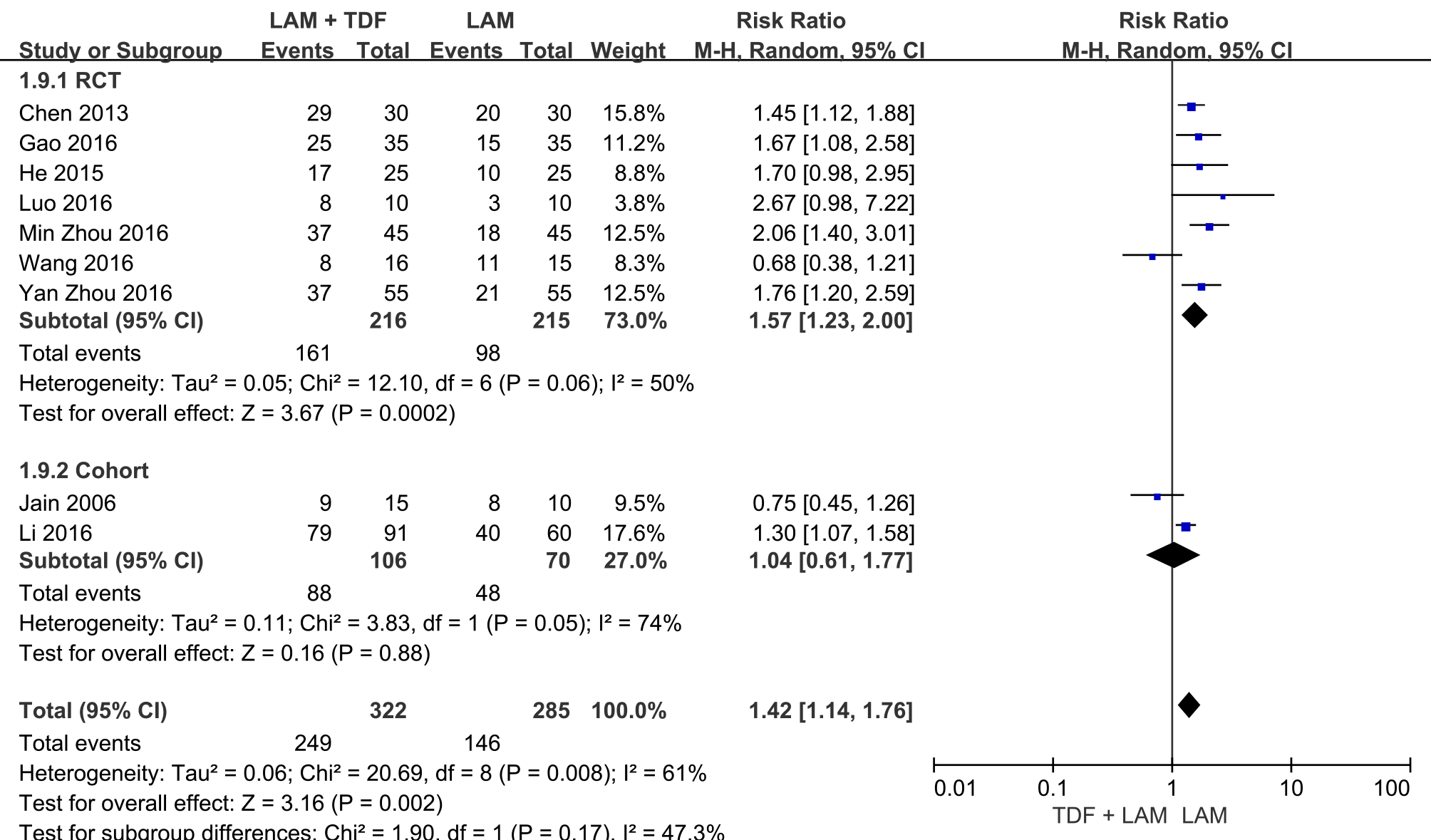

Supplement: Supplementary file 2 — Figure S2. Subgroup analyses by study design. (TIF 7777 kb) [file 12985_2018_1050_MOESM2_ESM.tif]

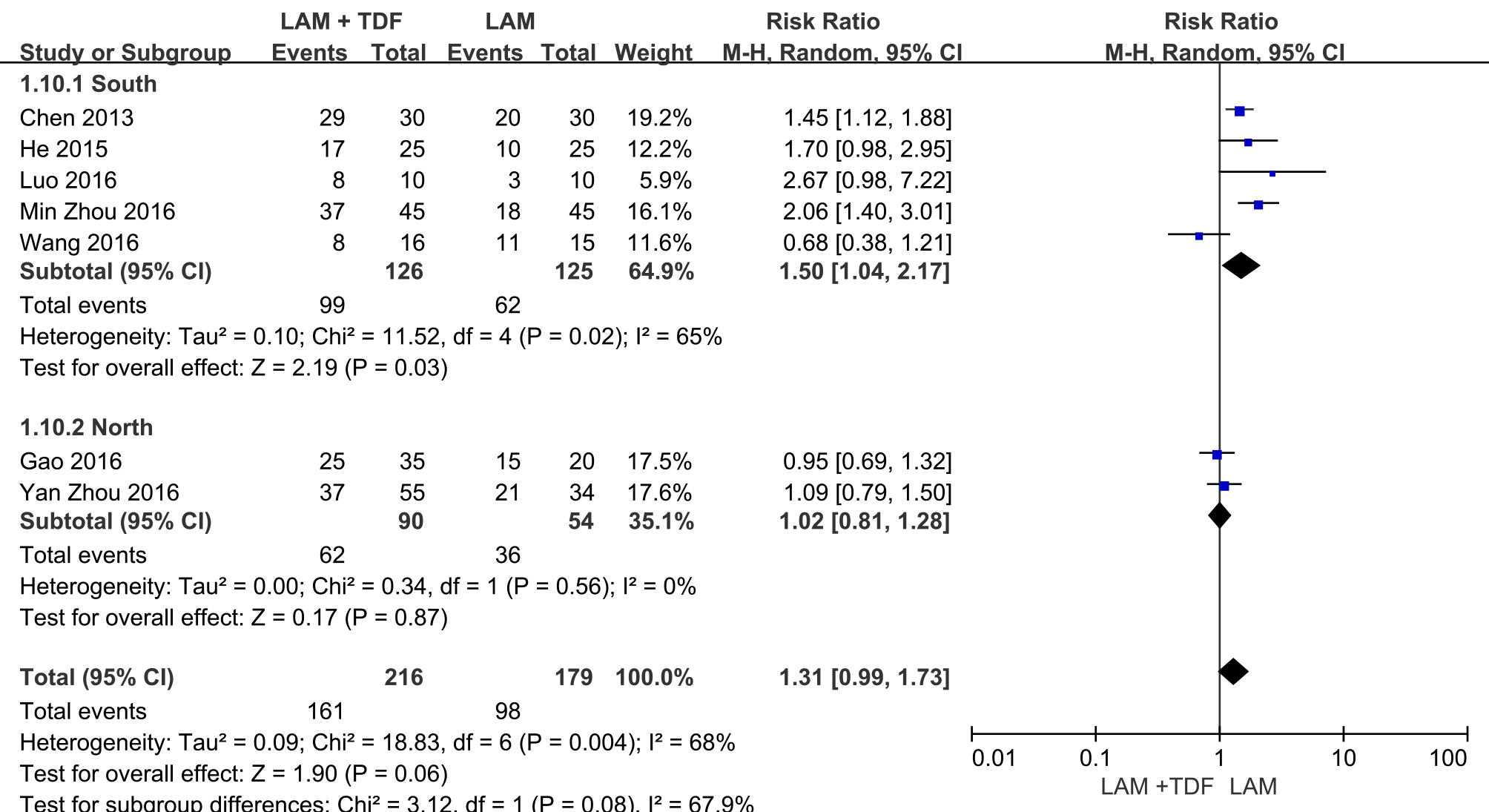

Supplement: Supplementary file 3 — Figure S3. Subgroup analyses by areas of China. (TIF 7222 kb) [file 12985_2018_1050_MOESM3_ESM.tif]

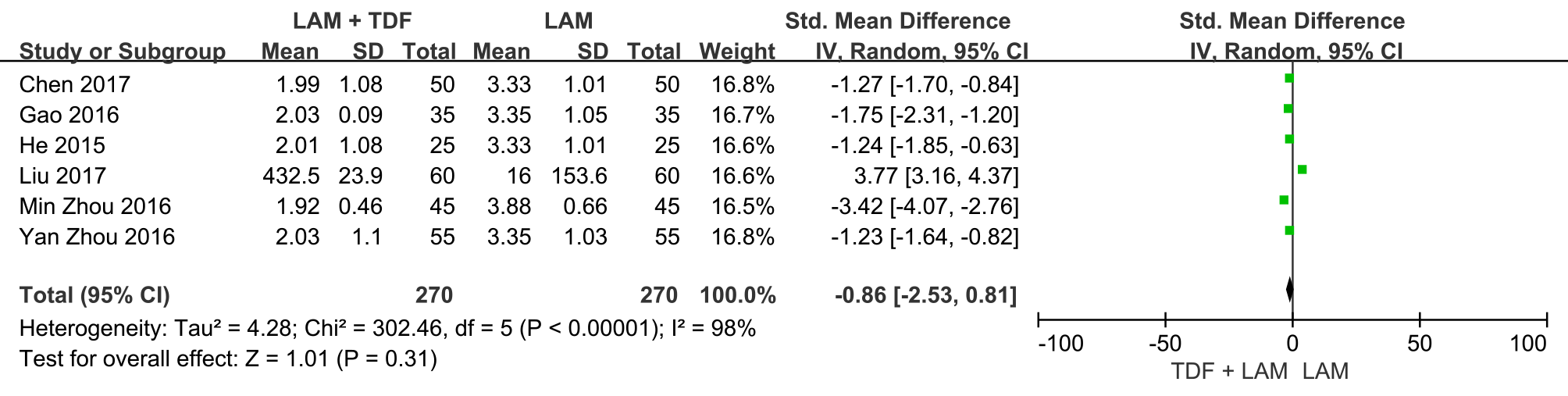

Supplement: Supplementary file 4 — Figure S4. Effect of LAM + TDF vs. LAM on HBV DNA levels at the end of treatment. (TIF 3430 kb) [file 12985_2018_1050_MOESM4_ESM.tif]

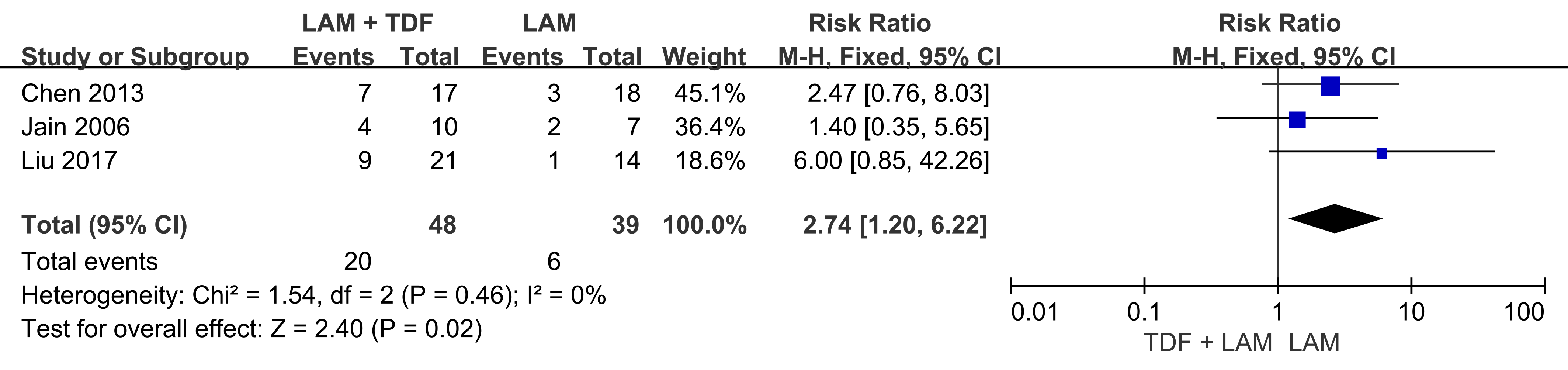

Supplement: Supplementary file 5 — Figure S5. Effect of LAM + TDF vs. LAM on the rate of HBeAg loss. (TIF 1169 kb) [file 12985_2018_1050_MOESM5_ESM.tif]

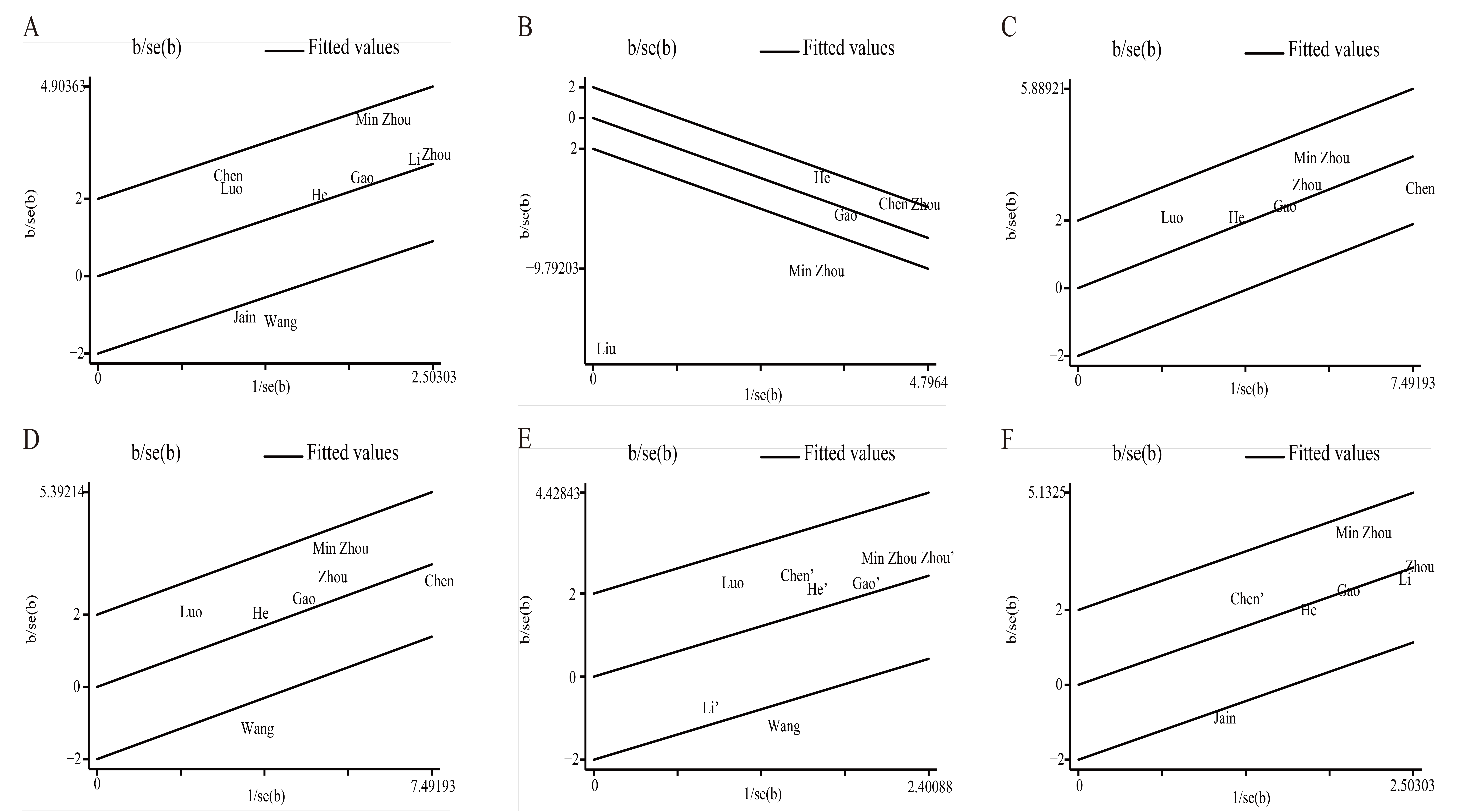

Supplement: Supplementary file 6 — Figure S6. Galbraith plots of HBV virological response rate(A), levels of HBV DNA(B), subgroups analysis of southern China(C), RCTs(D), 24 weeks(E), and 48 weeks(F). (TIF 6228 kb) [file 12985_2018_1050_MOESM6_ESM.tif]

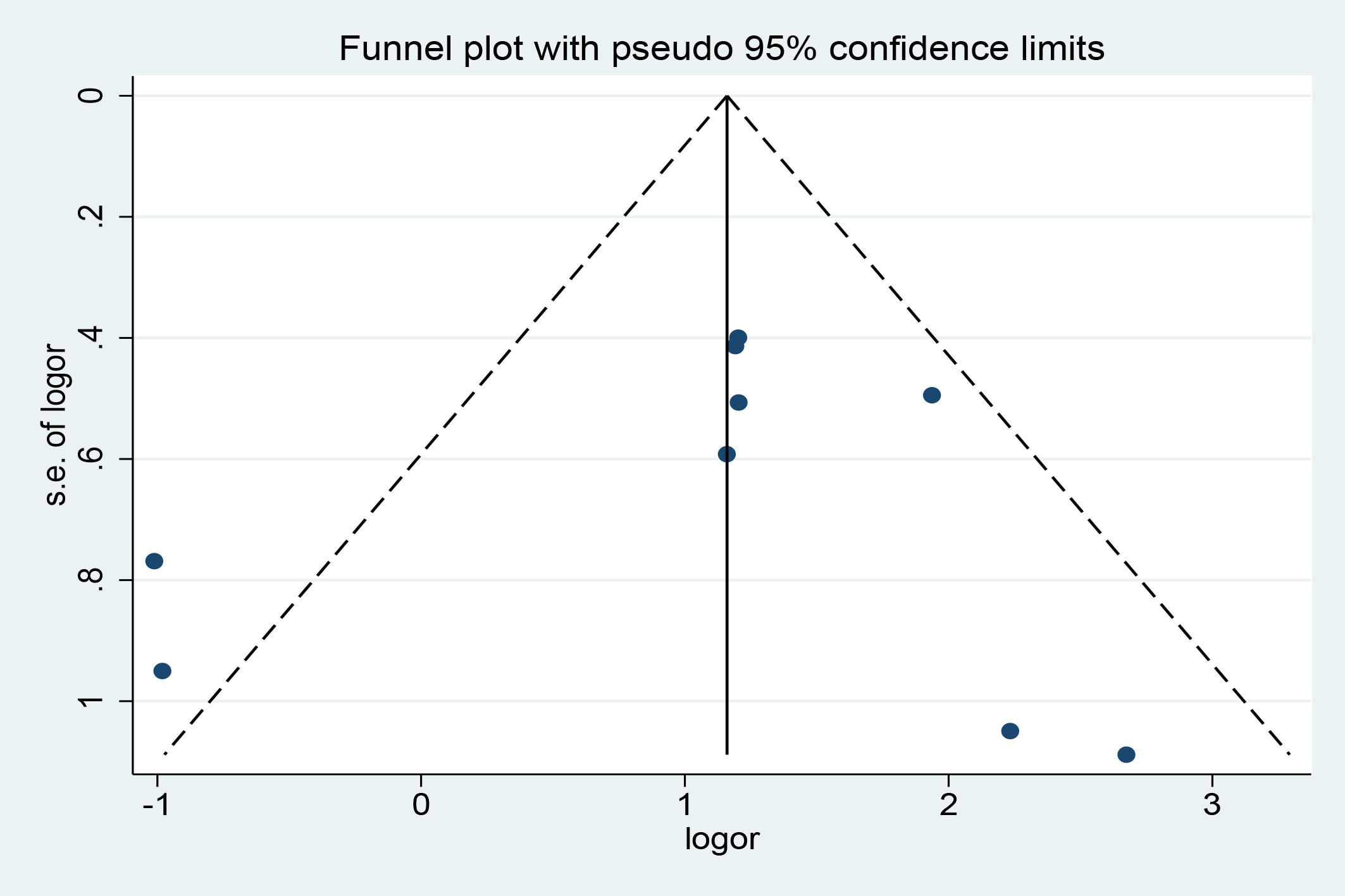

Supplement: Supplementary file 7 — Figure S7. Funnel plot for studies included for HBV virological responses. (TIF 516 kb) [file 12985_2018_1050_MOESM7_ESM.tif]
